# Synthesis and Preliminary Antimicrobial Activities of New Arylideneamino-1,3,4-thiadiazole-(thio/dithio)-acetamido Cephalosporanic Acids

**DOI:** 10.3390/molecules17011025

**Published:** 2012-01-19

**Authors:** Shakir Mahmood Alwan

**Affiliations:** Department of Pharmaceutical Chemistry, College of Pharmacy, University of Baghdad, Bab-Al-muadham P.O. Box 14026, 10047, Baghdad, Iraq; Email: shakmawales@yahoo.co.uk; Tel.: +00964-7902-518-888

**Keywords:** cephalosporins, 1,3,4-thiadiazole, antimicrobial activity, Schiff bases, disulfide bond

## Abstract

New derivatives of 7-aminocephalosporanic acid **1**–**8** were synthesized by acylation of the 7-amino group of the cephem nucleus with various arylidinimino-1,3,4-thiadiazole-thio(or dithio)-acetic acid intermediates **3a**–**d** and **5a**–**d**, respectively, so the acyl side chains of these new cephalosporins contained a sulfide or disulfide bond. This unique combination of a Schiff base with the sulfide or disulfide bonds in the acyl side chain afforded new cephalosporins of reasonable potencies, some of which were found to possess moderate activities against the tested microorganisms. Their chemical structures were characterized by ¹H-NMR, IR spectroscopy and elemental microanalysis. Preliminary *in vitro* antimicrobial activities of the prepared cephalosporins were investigated using a panel of selected microorganisms. Results indicated that the newly synthesized cephalosporins containing disulfide bonds (compounds **5**–**8**) exhibited better activities against *Staphylococcus aureus* and *Escherichia coli*. The cephalosporins cross-linked by a sulfide bond (compounds **1**–**4**) showed a slight change in antimicrobial activities when compared with that of the reference cephalosporin (cephalexin).

## 1. Introduction

Cephalosporin molecules containing heterocyclic moieties as part of the acyl side chain substituents at the C7 position have become increasingly popular in cephalosporin research [1,2,3,4]. These chemical modifications have led to the production of more potent cephalosporins. A successful cephalosporin containing a 1,3,4-thiadiazole moiety at the C3 position of the cephem nucleus showed a good improvement in activity against *Pseudomonas aeruginosa* and members of the *Enterobactericae* with higher serum levels [5,6]. However, certain cephalosporins containing a 1,2,4-thiadiazole moiety linked at the C7 acyl side chain were very potent, with improved MICs values [7,8,9]. The 1,3,4-thiadiazole and aminothiazole groups are associated with various biological activities, probably due to the presence of a toxophoric (-N=C-S-) group [9,10,11,12]. The importance of the sulfur atom in drugs as sulfide or disulfide linkages provides great stability for the three-dimensional structure of the molecule [13,14]. Besides, the presence of sulfur can have a great contributions to the antimicrobial activities [15,16]. Disulfide derivatives of 1,3,4-thiadiazole have shown antimicrobial activities against Gram (+) bacteria and fungi [17]. Schiff bases have also been widely reported as biologically versatile compounds having antifungal, herbicidal and plant growth regulating properties [18,19]. The presence of an isomethine group in certain compounds contributes to a large extent to the antimicrobial activities [19,20,21,22,23]. Compounds having Schiff’s bases demonstrated high resistance to β-lactamases and were very potent against members of the *Enterobacteriaceae* family [24,25].

To the best of the author’s knowledge, no cephalosporin resulting from coupling of 7-amino cephalosporanic acid with an acyl side chain containing an arylideneamino-1,3,4-thiadiazole linked by a disulfide bond has been reported so far.

β-Lactam antibiotics may be inactivated by bacterial enzymes (acylases and β-lactamases). Based on this fact, the development of this bacterial resistance has initiated the search for novel cephalosporins that may have reasonable potency and sensitivity against such microbes.

In view of these observations, new cephalosporins **1**–**8** containing 1,3,4-thiadiazole groups linked by either a sulfide or a disulfide bonds, in addition to a Schiff base, within the acyl side chain at C7 position have been designed, synthesized and characterized to be evaluated for their antimicrobial activities and compared with cephalexin as a reference.

## 2. Results and Discussion

### 2.1. Chemistry

**Scheme 1:**
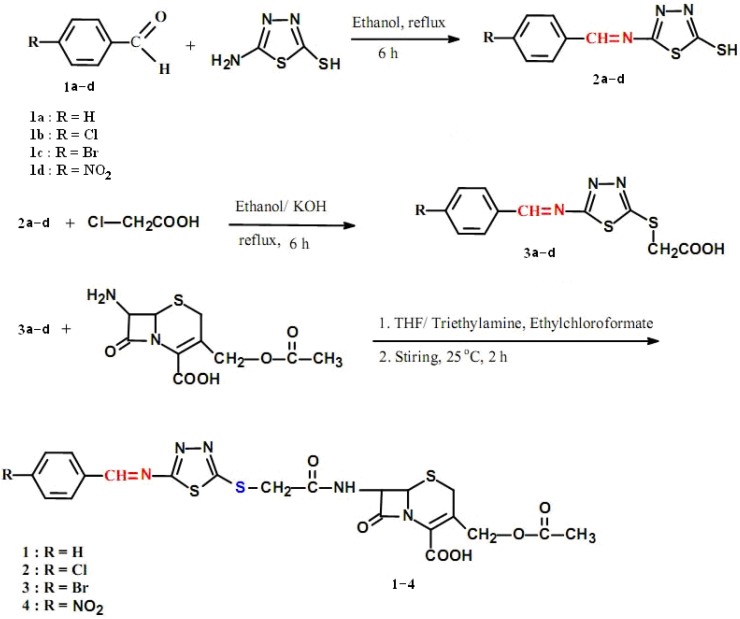
Synthesis of new arylideneamino-(1,3,4-thiadiazol-5-yl)-thioacetamido cephalosporanic acids.

**Scheme 2:**
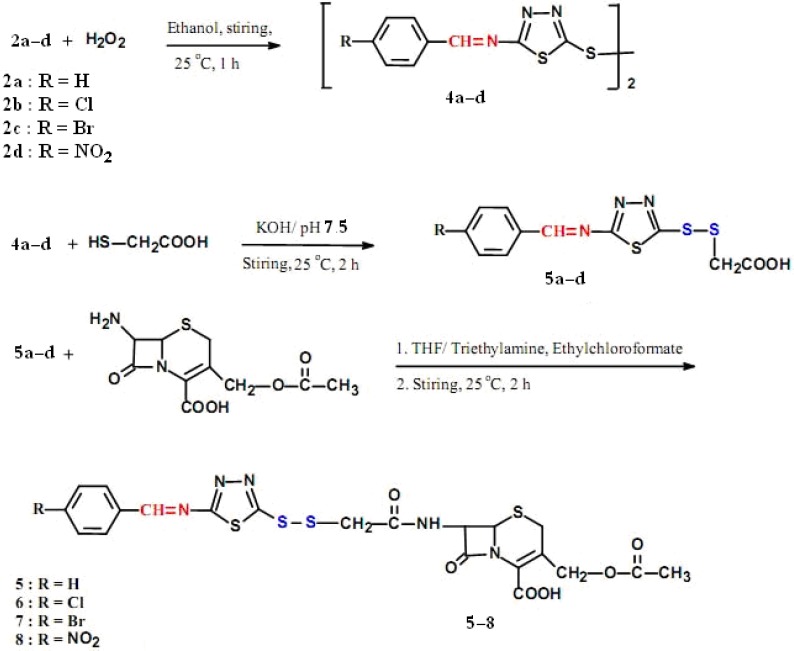
Synthesis of new arylideneamino-(1,3,4-thiadiazol-5-yl)-dithioacetamido cephalosporanic acids.

The designated compounds were synthesized according to Scheme 1 and Scheme 2. Two series of intermediates were synthesized as precursors of the target compounds by several experimental steps. In the first series, intermediates **2a**–**d** were synthesized by reacting the aromatic aldehydes **1a**–**d** with 2-amino-5-mercapto-1,3,4-thiadiazole in absolute ethanol using the method described previously [19], according to Scheme 1. This afforded Schiff bases **2a**–**d**. These Schiff bases **2a**–**d** were reacted with chloroacetic acid in an ethanolic KOH [26] whereupon new intermediates **3a**–**d** were obtained (Scheme 1). In the following step, **3a**–**d** were allowed to react with 7-amino cephalosporanic acid using the mixed anhydride method [27] leading to the formation of the arylideneamino-1,3,4-thiadiazolylthio-acetamidocephalosporanic acid target compounds **1**–**4** (Scheme 1).

These products were crystallized from ethanol. The chemical structures of these newly synthesized cephalosporins were confirmed by IR, ¹H-NMR spectral measurements and elemental microanalysis and were in good agreement with the proposed structures. The IR spectra of **2a**–**d** showed stretching absorption bands from 2,590–2,595 cm^−^^1^, attributed to the -SH function. The stretching absorption band of the C=N function appeared in the 1,610–1,630 cm^−^^1^ region, while the absorption band due to NH_2_ has disappeared. The bands appearing at 730–750 cm^−^^1^ were for the C-S function, while the aromatic C=C bond appeared at 1,530–1,590 cm^−^^1^.

In the IR spectra of **3a**–**d**, the C=O and O-H stretching absorption bands were found at 1,730 cm^−^^1^ and 3,100–3,300 cm^−^^1^, respectively. The IR spectra of compounds **3a**–**d** indicated the absence of a free -SH absorption band and the appearance of C=N at 1,610–1,630 cm^−^^1^. The target compounds **1**–**4** showed the following characteristic stretching absorption bands: β-lactam C=O appeared at 1,765–1,785 cm^−^^1^; C=O amide appeared at 1,650–1,660 cm^−^^1^; the carboxylic acid group C=O appeared at 1,720–1,750 cm^−^^1^: the C=N- of the Schiff base appeared at 1610 cm^−^^1^ and the C=C appeared at 1,550–1,570 cm^−^^1^. The IR spectra of compounds **1**–**4** were as follows; compound **1**, showed the following characteristic absorption bands appeared at: 3,100 cm^−^^1^ for N-H vibration, 1,785 cm^−^^1^ for C=O stretching vibration of the β-lactam, 1,750 cm^−^^1^ for COOH, 1,660 cm^−^^1^ for the amide stretching vibration, 1,630 cm^−^^1^ for C=N, 1,595 cm^−^^1^ for C=C aromatic and 1,175 cm^−^^1^ for C-S. Compounds **2** and **3** displayed identical absorption bands, besides, the presence of a band at 870 cm^−^^1^ for C-Cl and at 780 cm^−^^1^ for C-Br. However, compound **4** showed a similar pattern, in addition to bands at 1,525 cm^−^^1^ and 1,350 cm^−^^1^ for asymmetric and symmetric vibrations of NO_2_, respectively.

In the ¹H-NMR spectra of compounds **1**–**4** (60 MHz, DMSO-d_6_), the proton signals δ (ppm) were recorded as follows: 2.21(s, 3H, -CH_3_), 3.06–3.16 (m, 2H, -C4-H), 4.75(s, 2H, -CH_2_-O-CO), 5.55 (dd, 1H, C7-H), 5.10 (d, 1H, C6-H), 3.59 (s, 2H, -S-CH_2_-), 7.52–7.83 (m, 4H, Ar-H) or (m, 5H, Ar-H), 8.23 (d, 1H, -CO-NH-), 8.36 (s, 1H, -CH=N-), 12.56(s, 1H, -COOH).

Moreover, the elemental microanalysis results were all in good agreement with the proposed chemical structures for compounds **1**–**4**.

The second series of arylideneamino-1,3,4-thiadiazolyl-dithioacetamidocephalosporanic acids (compounds **5**–**8**) were synthesized using **2a**–**d**, as illustrated in Scheme 2. These intermediates **2a**–**d** were oxidized by hydrogen peroxide [28] leading to the formation of **4a**–**d**. Compounds **4a**–**d** are the disulfide-containing compounds prepared as symmetric bi-thiadiazolyl-2-arylideneamine derivatives. The IR spectra of **4a**–**d** were characterized by the disappearance of the free –SH absorption band and confirmation of the presence of absorption bands of -C=N at 1,610–1,630 cm^−^^1^. 

Compounds **5a**–**d** were prepared from **4a**–**d** through the sulfhydryl-disulfide exchange reaction using mercaptoacetic acid as previously reported [29,30]. Their chemical structures were confirmed by their IR spectra and chemical properties due to the appearance of free -COOH groups making compounds **5a**–**d** soluble in aqueous alkaline solutions. All of the above compounds **5a**–**d** containing Schiff bases and free carboxyl groups **5a**–**d** were reacted with 7-aminocephalosporanic acid using the mixed anhydride method [27] leading to the formation of the target cephalosporins (compounds **5**–**8**), as illustrated in Scheme 2.

The chemical structures of the newly synthesized cephalosporins **5**–**8** were confirmed by IR, ¹H-NMR spectral measurements and elemental microanalysis and were identical and very closely related to the measurements of compounds **1**–**4** as shown in Scheme 2. Due to the great similarity between their chemical structures there were only marginal differences in those measurements, in fact the only difference between these two series of compounds is the presence of disulfide bonds in compounds **5**–**8**, instead of sulfide bonds present in compounds **1**–**4**. However, compounds **5**–**8** exhibited IR and ¹H-NMR spectra consistent with their assigned structures, described in details in the Experimental section. The IR spectrum of compound **5** displayed the presence of the general characteristic absorption bands: β-lactam C=O appeared at 1,775, carboxyl C=O at 1,735, amide C=O at 1,710, C=N- at 1630, aromatic C=C at 1,575, C-S at 1,165 cm^−1^. Compound **6** showed similar bands as **5**, but in addition it showed a band at 870 cm^−^^1^ for C-Cl. Compound **7** displayed the C-Br at 780 cm^−^^1^, while compound **8** showed C-NO_2_ at 1520 cm^−^^1^ and 1335 cm^−^^1^ for asymmetric and symmetric vibrations, respectively.

In the ¹H-NMR of compounds **5**–**8**, the following peaks appeared: 2.21 (s, 1H, -CH_3_), 3.06–3.16 (m, 2H, C4-H), 3.58 (s, 2H, -S-CH_2_-), 4.75 (s, 2H, -CH_2_-O-C=O), 5.45 (dd, 1H, -C7-H), 5.10 (d, 1H, C6-H), 7.52–7.83 (m, 4H, Ar-H), 8.32 (d, 1H, -CO-NH), 8.36 (s, 1H, -N=CH-), 12.56 (s, 1H, -COOH). 

Elemental microanalysis results were within ± 0.4% of the theoretical values and in good agreement with the proposed chemical structures. The detailed data for all compounds are given in the Experimental section.

### 2.2. Antimicrobial Activities

The antimicrobial activities of the newly synthesized cephalosporins and cephalexin were evaluated by the agar diffusion method [31] using representative standard strains of Gram (+) and Gram (−) bacteria on tryptic soya agar media, as listed in Table 1. Dimethylsulfoxide was used as solvent for the test compounds. The minimum inhibitory concentrations (MIC values) of these cephalosporins were also determined using the standard dilution method [32].

Cephalosporins containing a 1,3,4-thiadiazole moiety linked through a disulfide bond in the acyl side chain compounds **5**–**8** were the most potent and were found to be equipotent to cephalexin, especially compounds **7** and **8**. This finding was expected and was supported when compared with antibiotics containing disulfide bonds which showed marked activities [17].

**Table 1:** Antimicrobial activities of the newly synthesized cephalosporins.

Compound	Antimicrobial Activity (zone of inhibition mm)			
	*M. letus* ATCC 9341	*S. aureus* ATCC 25923	*E. coli* ATCC 29522	*P. aeruginosa* ATCC27853
**1**	18	17	16	-
**2**	19	17	18	13
**3**	18	16	16	15
**4**	17	20	18	17
**5**	21	22	20	-
**6**	25	21	19	16
**7**	25	24	21	16
**8**	25	23	24	17
**Cephalexin**	26	22	20	-

## 3. Experimental

### 3.1. General

Melting points were determined in open capillary tubes on a Gallenkamp melting point apparatus and are uncorrected. The IR spectra were recorded (in KBr) on a Buck M500 model IR spectrophotometer and the wave numbers are given in cm^−^^1^. The ¹H-NMR spectra were recorded in DMSO-d_6_ solutions on Perkin Elmer 60 MHz spectrometer at ambient temperature. Chemical shifts were recorded in parts per million (ppm) and were referenced to tetramethylsilane. Elemental microanalyses were determined on a Carlo-Erba analyzer type 1106. Purity of the synthesized compounds was checked by TLC aluminium sheets coated with silica gel 60 F_254_ (0.2 mm thickness) and by melting points. 7-Aminocephalosporanic acid was a kind gift from Al-Nejma Bulk Pharmaceutical Company, Jordan. Benzaldehyde (**1a**), *p*-chlorobenzaldehyde (**1b**), *p*-bromo-benzaldehyde (**1c**), *p*-nitrobenzaldehyde (**1d)** and 2-amino-5-mercapto-1,3,4-thiadiazole were from Fluka. All solvents were Analar grade.

### 3.2. Chemical Methods

#### 3.2.1. General procedure for the preparation of 5-(arylideneamino)-1,3,4-thiadiazole-2-thiols **2a–d**

2-Amino-5-mercapto-1,3,4-thiadiazole was refluxed with the appropriate aldehyde **1a**–**d** in absolute ethanol for 6 h. The reaction mixture was cooled and the volume was reduced by vacuum and the products were precipitated, filtered, washed thoroughly with distilled water and crystallized from ethanol. Products were dried in an oven at 40–45 °C to give **2a**–**d**.

*5-(Benzylideneamino)-1,3,4-thiadiazole-2-thiol* (**2a**). This compound was prepared by reacting 2-amino-5-mercapto-1,3,4-thiadiazole (0.01 mol, 1.33 g) with benzaldehyde (**1a**, 0.01 mol, 1.06 g) in absolute ethanol (50 mL). Yield: 85%, m.p. 88–89 °C; IR (*v*, cm^−^^1^): 2590 (-SH), 1620 (C=N-), 1595 (C=C aromatic); ¹H-NMR δ (ppm): 13.0 (s, 1H, -SH), 8.36 (s, 1H, -CH=N-), 7.52–7.83 (m, 5H, Ar-H).

*5-(4-Chlorobenzylideneamino)-1,3,4-thiadiazole-2-thiol* (**2b**). 2-Amino-5-mercapto-1,3,4-thiadiazole (0.01 mol, 1.33 g) was reacted with 4-chlorobenzaldehyde (**1b**, 0.01 mol, 1.405 g) in absolute ethanol (50 mL). Yield: 80%, m.p. 180–182 °C; IR (*v*, cm^−^^1^): 2595 (-SH), 1630 (C=N-), 1590 (C=C aromatic), 875 (C-Cl); ¹H-NMR δ (ppm): 13.0 (s, 1H, -SH), 8.32 (s, 1H, -CH=N-), 7.52–7.77 (m, 4H, Ar-H).

*5-(4-Bromobenzylideneamino)-1,3,4-thiadiazole-2-thiol* (**2c**). Compound **2c** was prepared by reacting 2-amino-5-mercapto-1,3,4-thiadiazole (0.01 mol, 1.33 g) with 4-bromobenzaldehyde (**1c**, 0.01 mol, 1.45 g) in absolute ethanol (50 mL). Yield: 82%, m.p. 120–122 °C; IR (*v*, cm^−^^1^): 2595 (-SH), 1620 (C=N-), 1595 (C=C aromatic), 785 (C-Br); ¹H-NMR δ (ppm): 13.0 (s, 1H, -SH), 8.36 (s, 1H, -CH=N-), 7.52–7.77 (m, 4H, Ar-H). 

*5-(4-Nitrobenzylideneamino)-1,3,4-thiadiazole-2-thiol* (**2d**). This Schiff base was prepared as previously described using 2-amino-5-mercapto-1,3,4-thiadiazole (0.01 mol, 1.33 g) and 4-nitro-benzaldehyde (**1d**, 0.01 mol, 1.51 g) in absolute ethanol (50 mL). Yield: 88%, m.p. 98–100 °C; IR (*v*, cm^−^^1^): 2595 (-SH), 1620 (C=N-), 1600 (C=C aromatic), 1525 and 1350 (NO_2_); ¹H-NMR δ (ppm): 13.0 (s, 1H, -SH), 8.32 (s, 1H, -CH=N-), 8.09–8.33 (m, 4H, Ar-H).

#### 3.2.2. General procedure for the preparation of 2-(5-(arylideneamino)-1,3,4-thiadiazol-2-ylthio)acetic acids **3a–d**

These intermediates were prepared through the S-alkylation reactions using chloroacetic acid [26] to alkylate the sulfhydryl group of **2a**–**d** (Scheme 1) as follows: to a solution of **2a**–**d** (14.7 mmol) in ethanol (30 mL), potassium hydroxide (14.7 mmol) and chloroacetic acid (14.7 mmol) were added and the mixture was refluxed for 2 h. The hot mixture was filtered and the ethanolic solution was evaporated under reduced pressure. The residue was dissolved in distilled water, acidified with diluted hydrochloric acid to pH 3. The precipitate was collected by filtration, washed with cold distilled water and dried in an oven at 40–45 °C to provide **3a**–**d**. These products were crystallized from ethanol/water (1:1).

*2-(5-(Benzylideneamino)-1,3,4-thiadiazole-2-ylthio)acetic acid* (**3a**). Yield: 66%, m.p. 201–203 °C; IR (*v*, cm^−^^1^): 1750 (C=O), 1620 (C=N-), 1600 (C=C aromatic); ¹H-NMR δ (ppm): 12.59 (s, 1H, -COOH), 8.36 (s, 1H, -N=CH-), 7.52–7.83 (m, 5H, Ar-H), 4.15 (s, 2H,-CH_2_-).

*2-(5-(4-Chlorobenzylideneamino)-1,3,4-thiadiazole-2-ylthio)acetic acid* (**3b**). Yield: 58%, m.p. 244–246 °C; IR (*v*, cm^−^^1^): 1755 (C=O), 1630 (C=N-), 1600 (C=C aromatic), 870 (C-Cl); ¹H-NMR δ (ppm): 12.59 (s, 1H, -COOH), 8.32 (s, 1H,-CH=N-), 7.52–7.78 (m, 4H, Ar-H), 4.16 (s, 2H, -CH_2_-).

*2-(5-(4-Bromobenzylideneamino)-1,3,4-thiadiazole-2-ylthio)acetic acid* (**3c**). Yield: 70%, m.p. 166–168 °C; IR (*v*, cm^−^^1^): 1750 (C=O), 1620 (C=N-), 1595 (C=C aromatic), 780 (C-Br); ¹H-NMR: δ (ppm); 12.59 (s, 1H, -COOH), 8.36 (s, 1H, -N=CH-), 7.58–7.72 (m, 4H, Ar-H), 4.16 (s, 2H, -CH_2_-).

*2-(5-(4-Nitrobenzylideneamino)-1,3,4-thiadiazole-2-ylthio)acetic acid* (**3d**). Yield: 60%, m.p. 194–196 °C; IR (*v*, cm^−^^1^): 1745 (C=O), 1620 (C=N-), 1595 (C=C aromatic), 1525 and 1350 (NO_2_); ¹H-NMR δ (ppm); 12.59 (s, 1H, -COOH), 8.33 (s, 1H, -N=CH-), 8.09–8.33 (m, 4H, Ar-H), 4.14 (s, 2H, -CH_2_-).

#### 3.2.3. General procedure for the preparation of 3-(acetoxymethyl)-7-(2-(5-(arylideneamino)-1,3,4-thiadiazol-2-ylthio)acetamido)-8-oxo-5-thia-1-aza-bicyclo[4.2.0]oct-2-ene-2-carboxylic acids **1–4**

These new cephalosporins were prepared using the mixed anhydride method [27], as illustrated in Scheme 1. An anhydrous solution of **3a**–**d** (11.4 mmol) and triethylamine (11.4 mmol) in THF (30 mL) was cooled to −10 °C. To this solution, an aliquot of ethylchloroformate (11.4 mmol) was added dropwise with continuous stirring. The resulting mixture was left for 30 min. with continuous stirring at 0 °C. A cold aqueous solution (10 mL) of 7-aminocephalosporanic acid (11.4 mmol) and triethylamine (11.4 mmol) was added to the above mixture. The final mixture was vigorously stirred for 2 h at room temperature, diluted with distilled water (30 mL) and then was extracted with diethyl ether (2 × 20 mL). The aqueous phase was acidified with dilute HCl to pH 3, and extracted with ethyl acetate (3 × 20 mL). The extracts were pooled together, dried over anhydrous sodium sulfate and then evaporated to dryness under reduced pressure. The residue was triturated with petroleum ether, crystallized from ethanol and collected to afford compounds **1**–**4**.

*3-(Acetoxymethyl)-7-(2-(5-(benzylideneamino)-1,3,4-thiadiazol-2-ylthio)acetamido)-8-oxo-5-thia-1-azabicyclo[4.2.0]oct-2-ene-2-carboxylic acid* (**1**). Yield: 55%, m.p. 176 °C (decomposed); Anal. % calcd. for C_21_H_19_N_5_O_6_S_3_ (533.6); C: 47.27, H: 3.59, N: 13.12; found: C: 47.04, H: 3.54, N: 13.06; IR spectra (*v*, cm^−^^1^): 3,100 (N-H), 1,785 (β-lactam C=O), 1,750 (C=O), 1,660 (amide C=O), 1,630 (C=N), 1,595 (C=C aromatic); ¹H-NMR δ (ppm): 12.56 (s, 1H, -COOH), 8.36 (s, 1H, -N=CH-), 8.23 (d, 1H, -CO-NH-), 7.52–7.83 ( m, 5H, Ar-H), 5.56 (d, 1H, C7-H), 5.10 (dd, 1H, -C6-H), 4.75 (s, 2H, -CH_2_-O-C=O), 3.56 (s, 2H, -S-CH_2_- ), 3.09–3.16 (m , 2H, C4-H), 2.23 (s, 3H, -CH_3_).

*3-(Acetoxymethyl)-7-(2-(5-(4-chlorobenzylideneamino)-1,3,4-thiadiazol-2-ylthio)acetamido)-8-oxo-5-thia-1-azabicyclo[4.2.0]oct-2-ene-2-carboxylic acid* (**2**). Yield: 58%, m.p. 142–144 °C. Anal. % calcd. for C_21_H_18_N_5_O_6_S_3_Cl (568.0); C: 44.40, H: 3.19, N: 12.33; found: C: 44.23, H: 3.16, N: 12.29; IR (*v*, cm^−^^1^): 3,150 (N-H), 1,780 (β-lactam C=O), 1,750 (C=O), 1,730 (amide C=O), 1,630 (C=N group), 1,605 (C=C aromatic), 870 (C-Cl); ¹H-NMR δ (ppm): 12.56 (s, 1H, -COOH), 8.36 (s, 1H, -CH=N-), 8.23 (d , 1H, -CO-NH- ), 7.52–7.83 (m, 4H, Ar-H), 5.75 (d, 1H, C7-H ), 5.10 (dd, 1H, -C6-H), 4.75 (s, 2H,-CH_2_-O-C=O), 3.56 (s, 2H, -S-CH_2_-), 3.09–3.16 (m , 2H, C4-H), 2.23 (s, 3H, -CH_3_).

*3-(Acetoxymethyl)-7-(2-(5-(4-bromobenzylideneamino)-1,3,4-thiadiazol-2-ylthio)acetamido)-8-oxo-5-thia-1-aza-bicyclo[4.2.0]oct-2-ene-2-carboxylic acid* (**3**). Yield: 60%, m.p. 133 °C (decomposed). Anal. % calcd. for C_21_H_18_N_5_O_6_S_3_Br (612.5); C: 41.18, H: 2.96, N: 11.43; found: C: 41.34, H: 2.99, N: 11.47. IR (v, cm^−^^1^): 1,785 (β-lactam C=O), 1,720 (amide C=O), 1,630 (C=N), 1,745 (C=O), 1,600 (C=C aromatic), 3,150 (N-H), 780 (C-Br); ¹H-NMR δ (ppm): 12.56 (s,1H, -COOH), 8.36 (s, 1H, -CH=N-), 7.95 (d , 1H, -CO-NH-), 7.52–7.72 ( m, 4H, Ar-H), 5.56 (d, 1H, C7-H ), 5.10 (dd, 1H, -C6-H), 4.70 (s, 2H, -CH_2_-O-), 3.56 (s, 2H, -S-CH_2_-), 3.09–3.16 (m , 2H, C4-H), 2.23 (s, 3H, -CH_3_).

*3-(Acetoxymethyl)-7-(2-(5-(4-nitrobenzylideneamino)-1,3,4-thiadiazol-2-ylthio)acetamido)-8-oxo-5-thia-1-aza-bicyclo[4.2.0]oct-2-ene-2-carboxylic acid* (**4**). Yield: 45%, m.p. 148 °C (decomposed). Anal. % calcd. for C_21_H_18_N_6_O_8_S_3_ (578.6); C: 43.59, H: 3.14, N: 14.52; found: C: 43.42, H: 3.09, N: 14.48. IR (v, cm^−^^1^); 1,785 (β-lactam C=O), 1,745 (carboxylate C=O), 1,725 (amide C=O), 1,630 (C=N), 1,528 and 1,350 (NO_2_), 3,150 (N-H), 870 (C-NO_2_); ¹H-NMR δ (ppm): 12.56 (s, 1H, -COOH), 8.31 (s, 1H, -CH=N-), 8.23 (d, 1H, -CO-NH-), 8.09–8.33 (m, 4H, Ar-H), 5.56 (d, 1H, C7-H ), 5.10 (dd, 1H, -C6-H), 4.75 (s, 2H,-CH_2_-O-C=O), 3.45 (s, 2H, -S-CH_2_- ), 3.09–3.16 (m, 2H, C4-H), 2.23 (s, 3H, -CH_3_).

#### 3.2.4. General procedure for the preparation of 5,5'-disulfanyl-bis-(N-arylideneamino-1,3,4-thiadiazoles) **4a–d**

These intermediates were prepared by oxidizing **2a**–**d** using hydrogen peroxide [28] (Scheme 2) as follows: Hydrogen peroxide (14.8 mmol) was added dropwise to a solution of **2a**–**d** (22 mmol) in absolute ethanol (50 mL) with continuous stirring for 1 hr at room temperature. A white precipitate was obtained, collected by filtration, washed excessively with distilled water and dried in an oven at 40 °C. Products were crystallized from ethanol.

*N-Benzylidene-5-(2-(benzylideneamino)-1,3,4-thiadiazol-2-yl)disulfanyl)-1,3,4-thiadiazol-2-amine* (**4a**). Yield: 46%, m.p. 159–161 °C. IR (*v*, cm^−^^1^): 1,620 (C=N), 1,580 (C=C aromatic); ¹H-NMR δ (ppm): 8.35 (s, 2H, 2-CH=N-), 7.52–7.83 (m, 10H, Ar-H).

*N-Benzylidene-5-(2-(4-chlorobenzylideneamino)-1,3,4-thiadiazol-2-yl)disulfanyl)-1,3,4-thiadiazol-2-amine* (**4b**). Yield: 43%, m.p. 216–218 °C. IR (*v*, cm^−^^1^): 1,630 (C=N), 1,595 (C=C aromatic), 895 (C-Cl); ¹H-NMR δ (ppm): 8.36 (s, 2H, 2-CH=N-), 7.52–7.78 (m, 8H, Ar-H).

*N-Benzylidene-5-(2-(4-bromobenzylideneamino)-1,3,4-thiadiazol-2-yl)disulfanyl)-1,3,4-thiadiazol-2-amine* (**4c**). Yield: 42%, m.p. 227–229 °C. IR (*v*, cm^−^^1^): 1,610 (C=N), 1,575 (C=C aromatic), 790 (C-Br); ¹H-NMR δ (ppm): 8.36 (s, 2H, 2-CH=N-), 7.52–7.83 (m, 8H, Ar-H).

*N-Benzylidene-5-(2-(4-nitrobenzylideneamino)-1,3,4-thiadiazol-2-yl)disulfanyl)-1,3,4-thiadiazol-2-amine* (**4d**). Yield: 45%, m.p. 178–180 °C. IR (*v,* cm^−^^1^): 1,600 (C=N), 1,585 (C=C aromatic), 1,510 and 1,350 (NO_2_); ¹H-NMR δ (ppm): 8.36 (s, 2H, 2-CH=N-), 8.09–8.33 (m, 8H, Ar-H).

#### 3.2.5. General procedure for the preparation of (*E*)-2-(2-(5-(arylideneamino)-1,3,4-thiadiazol-2-yl)disulfanyl) acetic acids **5a–d**

A thiol–disulfide exchange reaction [29,30] was employed in the preparation of these intermediates, as illustrated in Scheme 2. An aqueous solution (30 mL) containing mercaptoacetic acid (7.4 mmol) and potassium hydroxide (7.4 mmol) adjusted to pH 7.5 was added to a suspension of **4a**–**d** (7.4 mmol) in potassium chloride (2 M, 25 mL) at pH 7.5. The mixture was vigorously stirred for 2 hr and then acidified with dilute HCl. The precipitated product was collected by filtration, washed thoroughly with distilled water and dried in an oven at 40 °C to afford **5a**–**d**.

*(E)-2-(2-(5-(Benzylideneamino)-1,3,4-thiadiazol-2-yl)disulfanyl) acetic acid* (**5a**). Yield: 83%, m.p. 226–228 °C. IR (*v*, cm^−^^1^): 1,725 (C=O), 1,635 (C=N), 1,575 (C=C); ¹H-NMR δ (ppm): 12.59 (s, 1H, -COOH), 8.36 (s, 1H, -CH=N-), 7.52–7.83 (m, 5H, Ar-H), 3.50 (s, 2H, -CH_2_-).

*(E)-2-(2-(5-(4-Chlorobenzylideneamino)-1,3,4-thiadiazol-2-yl)disulfanyl) acetic acid* (**5b**). Yield: 77%, m.p. 216–218 °C; IR (*v*, cm^−^^1^): 1,735 (C=O), 1,630 (C=N), 1,570 (C=C), 875 (C-Cl); ¹H-NMR δ (ppm): 12.56 (s, 1H, -COOH), 8.36 (s, 1H, -CH=N-), 7.52–7.77 (m, 4H, Ar-H), 3.5 (s, 2H, -CH_2_-).

*(E)-2-(2-(5-(4-Bromobenzylideneamino)-1,3,4-thiadiazol-2-yl)disulfanyl) acetic acid* (**5c**). Yield: 82%, m.p. 240–242 °C; IR (*v*, cm^−^^1^): 1,725 (C=O) 1,610 (C=N), 1,560 (C=C), 785 (C-Br): ¹H-NMR δ (ppm): 12.56 (s, 1H, -COOH), 8.36 (s, 1H, -CH=N-), 7.52–7.77 (m, 4H, Ar-H), 3.5 (s, 2H, -CH_2_-).

*(E)-2-(2-(5-(4-Nitrobenzylideneamino)-1,3,4-thiadiazol-2-yl)disulfanyl) acetic acid* (**5d**). Yield: 80%, m.p. 232–234 °C. IR (*v*, cm^−^^1^): 1,735 (carboxylate C=O), 1,635 (C=N), 1,595 (C=C aromatic), 1,525 and 1,340 (NO_2_); ¹H-NMR δ (ppm): 12.56 (s, 1H, -COOH), 8.36 (s, 1H, -CH=N-), 8.09–8.33 (m, 4H, Ar-H), 3.5 (s, 2H, -CH_2_-).

#### 3.2.6. General procedure for the preparation of 3-(acetoxymethyl)-7-(2-((5-(arylideneamino)-1,3,4-thiadiazol-2-yl)disulfanyl)acetamido)-8-oxo-5-thia-1-azabicyclo[4.2.0]oct-2-ene-2-carboxylic acids **5–8**

These new cephalosporins containing disulfide bonds were prepared by reacting **5a**–**d** with 7-aminocephalosporanic acid using the mixed anhydride method 27 as previously described and shown in Scheme 2. The percent yield and m.p. are listed for each case. Elemental microanalysis and IR spectra for compounds **5**–**8** are presented for each compound separately.

*3-(Acetoxymethyl)-7-(2-((5)-benzylideneamino)-1,3,4-thiadiazol-2-yl)disulfanyl)acetamido)-8-oxo-5-thia-1-azabicyclo[4.2.0]oct-2-ene-2-carboxylic acid* (**5**). Yield: 55%, m.p. 262 °C (decomposed): Anal. % calcd. for C_21_H_19_N_5_O_6_S_4_ (565.7): C: 44.59, H: 3.39; N: 12.38; found: C: 44.37, H: 3.34, N: 12.32. IR (v, cm^−^^1^): 3,100 (N-H), 1,775 (β-lactam C=O), 1,735 (carboxylate C=O), 1,710 (amide C=O), 1,640 (C=N); ¹H-NMR δ (ppm): 12.56 (s, 1H, -COOH), 8.36 (s, 1H, -N=CH-), 8.32 (d, 1H, -CO-NH-), 7.52–7.83 (m, 5H, Ar-H), 5.45 (d, 1H, C7-H), 5.10 (s, 1H, -C6-H), 4.75 (s, 2H, -CH_2_-O-C=O), 3.45 (s, 2H, -S-CH_2_-), 3.06–3.16 (m, 2H, C4-H), 2.21 (s, 3H, -CH_3_).

*3-(Acetoxymethyl)-7-(2-((5-(4-chlorobenzylideneamino)-1,3,4-thiadiazol-2-yl)disulfanyl)acetamido)-8-oxo-5-thia-1-azabicyclo[4.2.0]oct-2-ene-2-carboxylic acid* (**6**). Yield: 60%, m.p. 232 °C (decomposed); Anal. % calcd. for C_21_H_18_N_5_O_6_S_4_Cl (600.1); C: 42.03, H: 3.02, N: 11. 67; found: C: 41.88, H: 2.99, N: 11.62. IR (v, cm^−^^1^): 3,100 (N-H), 1,765 (β-lactam C=O), 1,735 (carboxylate C=O), 1,640 (C=N), 870 (C-Cl); ¹H-NMR δ (ppm): 12.56 (s, 1H, -COOH), 8.36 (s, 1H, -N=CH-), 8.32 (d, 1H, -CO-NH-), 7.52–7.83 (m, 4H, Ar-H), 5.45 (d, 1H, C7-H), 5.10 (dd, 1H, -C6-H), 4.75 (s, 2H, -CH_2_-O-C=O), 3.50 (s, 2H, -S-CH_2_-), 3.06–3.16 (m, 2H, C4-H), 2.21 (s, 3H, -CH_3_).

*3-(Acetoxymethyl)-7-(2-((5-(4-bromobenzylideneamino)-1,3,4-thiadiazol-2-yl) disulfanyl) acetamido)-8-oxo-5-thia-1-azabicyclo[4.2.0]oct-2-ene-2-carboxylic acid* (**7**). Yield: 58%, m.p. 222 °C (decomposed); Anal. % calcd. for C_21_H_18_N_5_O_6_S_4_Br (644.6); C: 39.13, H: 2.81, N: 10.87; found: C: 39.28, H: 2.85, N: 10.91; IR (v, cm^−^^1^): 3,150 (N-H), 1,785 (β-lactam C=O), 1,785 (β-lactam C=O), 1,735 (carboxylat e C=O), 1,640 (C=N), 780 (C-Br); H-NMR δ (ppm): 12.56 (s, 1H, -COOH), 8.36 (s, 1H, -N=CH-), 8.32 (d, 1H, -CO-NH-), 7.52–7.83 (m, 4H, Ar-H), 5.45 (d, 1H, C7-H), 5.10 (dd, 1H, -C6-H), 4.75 (s, 2H, -CH_2_-O-C=O), 3.45 (s, 2H, -S-CH_2_-), 3.06–3.16 (m, 2H, C4-H), 2.21 (s, 3H, -CH_3_).

*3-(Acetoxymethyl)-7-(2-((5-(4-nitrobenzylideneamino)-1,3,4-thiadiazol-2-yl)disulfanyl)acetamido)-8-oxo-5-thia-1-azabicyclo[4.2.0]oct-2-ene-2-carboxylicacid* (**8**). Yield: 62%, m.p. 158 °C (decomposed); Anal. % calcd. for C_21_H_18_N_6_O_8_S_4_ (610.7); C: 41.30; H: 2.97; N: 13.76; found: C: 41.15; H: 2.94; N: 13.72. IR (v, cm^−^^1^): 3,100 (N-H), 1,780 (β-lactam C=O), 1,730 (carboxylate C=O), 1,620 (C=N), 1,520 and 1,335 (NO_2_); ¹H-NMR δ (ppm): 12.56 (s, 1H, -COOH), 8.36 (s, 1H, -N=CH-), 8.32 (d, 1H, -CO-NH-), 8.09–8.33 (m, 4H, Ar-H), 5.50 (d, 1H, C7-H), 5.10 (dd, 1H, -C6-H), 4.75 (s, 2H, -CH_2_-O-C=O), 3.45 (s, 2H, -S-CH_2_-), 3.06–3.16 (m, 2H, C4-H), 2.21 (s, 3H, -CH_3_).

### 3.3. Preliminary Antimicrobial Assay

The antimicrobial activities of the newly synthesized cephalosporins (compounds **1**–**8**) were determined by the agar diffusion method 31 using representative Gram (+) and Gram (−) bacteria on tryptic soya agar media. The test microorganisms used to evaluate the potential antimicrobial activity of the newly synthesized cephalosporins were: *Staphylococcus aureus* ATCC 25923, *Escherichia coli* ATCC 25922, *Pseudomonas auroginosa* ATCC 27853, *Micrococcus letus* ATCC 9341. The cephalosporins were dissolved in dimethylsulfoxide to prepare the test solutions of 1 µg/mL concentration. Cephalexin was used as a reference and the activities were presented as zones of inhibition for each compound (Table 2).

**Table 2:** Minimum inhibitory concentrations of the newly synthesized cephalosporins (MIC values, μg/mL).

Compound	*M. letus* ATCC 9341	*S. aureus* ATCC 25923	*E. coli* ATCC 29522	*P. aeruginosa* ATCC27853
**1**	34	32	50	Not Tested
**2**	33	35	38	88
**3**	30	34	38	90
**4**	28	30	36	92
**5**	25	30	38	Not Tested
**6**	26	28	28	82
**7**	16	17	25	82
**8**	13	23	24	80
**Cephalexin**	15	25	30	Not Tested

Determination of the minimum inhibitory concentrations (MIC) was also achieved using the standard two-fold dilution method [32]. A suspension of various microorganisms from sterile overnight cultures on Tryptic soya broth media was prepared by dilution with sterile tryptic soya broth (1:100). The antimicrobial assay results (MIC values) are presented on Table 2.

## 4. Conclusions

Spectral and analytical data of the newly synthesized cephalosporins were all in good agreement with the proposed chemical structures. The antimicrobial activities of the synthesized compounds **1**–**8** were recorded against certain microbes and were reasonable when compared with cephalexin. It was concluded that the incorporation of a 1,3,4-thiadiazole moiety through a disulfide bond within the acyl side chain of cephalosporanic acid produced compounds **5**–**8** with comparable activity and relatively more potent than those compounds containing sulfide bonds (**1**–**4**). Generally, they were equipotent with the reference cephalexin. Further studies are in progress to obtain more information about these new cephalosporins, especially against resistant strains of microbes.
